# MiR-503-5p alleviates peripheral neuropathy-induced neuropathic pain in T2DM mice by regulating SEPT9 to inhibit astrocyte activation

**DOI:** 10.1038/s41598-024-65096-z

**Published:** 2024-06-21

**Authors:** Yuqing Guo, Jingyang Zeng, Yuanzhao Zhuang, Changcheng Jiang, Wenqin Xie

**Affiliations:** Department of Anesthesiology, Quanzhou First Hospital Affiliated to Fujian Medical University, No. 250, East Street, Licheng District, Quanzhou, 362800 Fujian China

**Keywords:** miR-503-5p, SEPT9, Diabetic peripheral neuropathy, T2DM mice, Astrocytes, Immunology, Neurology

## Abstract

Diabetic peripheral neuropathy (DPN) is a common complication of type 2 diabetes mellitus (T2DM) that causes peripheral and autonomic nervous system dysfunction. Dysregulation of miRNAs plays a crucial role in DPN development. However, the role of miR-503-5p in DPN remains unknown. Herein, T2DM mice (db/db) were used as a DPN model in vivo, and astrocytes isolated from db/db mice were induced with high glucose levels as a DPN model in vitro. MiR-503-5p expression was analyzed using qRT-PCR. GFAP, MCP-1, and SEPT9 protein levels were analyzed using western blotting and immunofluorescence. Luciferase assays were performed to investigate the interaction between miR-503-5p and SEPT9. We found that miR-503-5p expression decreased in the spinal cord of DPN model mice and astrocytes treated with high glucose (HG). The db/db mice displayed higher body weight and blood glucose, lower mechanical withdrawal threshold and thermal withdrawal latency, and higher GFAP and MCP-1 protein levels than db/m mice. However, tail vein injection of agomiR-503-5p remarkably reversed these parameters, whereas antigomiR-503-5p enhanced them. HG markedly facilitated GFAP and MCP-1 protein expression in astrocytes, whereas miR-503-5p mimic or inhibitor transfection markedly blocked or elevated GFAP and MCP-1 protein expression, respectively, in astrocytes with HG. SEPT9 was a target of miR-503-5p. In addition, SEPT9 protein levels were found to be elevated in db/db mice and astrocytes treated with HG. Treatment with agomiR-503-5p and miR-503-5p mimic was able to reduce SEPT9 protein levels, whereas treatment with antigomiR-503-5p and miR-503-5p inhibitor led to inhibition of the protein. Furthermore, SEPT9 overexpression suppressed the depressing effect of miR-503-5p overexpression in astrocytes subjected to HG doses. In conclusion, miR-503-5p was found to alleviate peripheral neuropathy-induced neuropathic pain in T2DM mice by regulating SEPT9 expression.

## Introduction

Diabetes mellitus (DM) is a fast-growing metabolic disease accompanied by various complications in later stages, posing a serious threat to patients’ quality of life and health^1^. Diabetic peripheral neuropathy (DPN) is a common complications of DM, which leads to the dysfunction of the peripheral and autonomic nervous systems to include spontaneous pain, hyperalgesia, and abnormal pain sensation^2^. Patients with DPN often experience symmetrical pain in their extremities, particularly distal pain^3^. Despite curative improvements in the therapeutic strategies for DPN, the efficacy and side effects of the current drugs used to prevent or treat DPN remain controversial. Thus, to ameliorate neuropathic pain in patients with DPN, it is crucial to develop novel targeted therapies that expand on the latent molecular mechanisms of DPN development. Astrocyte activation is associated with neuropathic pain^4^. Inhibition of astrocyte activation can alleviate pain caused by diabetic peripheral neuropathy^5,6^. Therefore, regulating astrocyte activation may improve neuropathic pain. However, the exact regulatory mechanisms underlying this process are not completely understood.

Recently, a growing number of researchers have suggested that miRNAs play a crucial role in the progression of DPN^7^, suggesting their potential diagnostic and clinical applications. For instance, Feng et al.^8^ showed that miR-146a levels were markedly blocked in rats with DPN and that miR-146a participates in the pathological process of DPN by regulating inflammatory responses. miR-7a-5p is expressed at low levels in DPN rats and is involved in RSC96 cell proliferation, apoptosis, calcium release, and oxidative stress response, whereas the miR-7a-5p mimic attenuates mitochondrial dysfunction in DPN by modulating VDAC1 expression^9^. The suppression of miRNA-155 can ameliorate sciatic nerve injury in DPN by alleviating inflammation^10^. As a multifunctional miRNA, miR-503-5p is involved in various tumor processes including colon cancer^11^ and hepatocellular carcinoma^12^. MiR-503-5p promotes inflammation, insulin resistance, and β-cell decompensation, improving β-cell function in aging mice^13^. miR-503-5p also plays an important role in the development of diabetic complications. In diabetic cardiomyopathy and foot syndrome, the expression of miR-503-5p is significantly upregulated^14,15^. In diabetic nephropathy, miR-503-5p enhances endoplasmic reticulum stress and cell apoptosis by downregulating BCL-2^16^. In high glucose-induced microvascular endothelial cell injury, miR-503-5p/Apelin-12 regulates inflammation and oxidative stress^17^. However, the role of miR-503-5p in DPN remains unknown.

SEPT9 is a highly conserved cytoskeletal protein that is widely found in eukaryotes and is involved in cell polarization, transport of substances inside or outside cells, and regulation of the cell cycle and apoptosis^18^. SEPT9 methylation affects cancer progression similar to that in glioma^19^ and gastric cancer^20^. SEPT9 expression is associated with neural pain caused by hereditary neuropathic atrophy^21^. In addition, SEPT9 is abnormally overexpressed in rats with DPN, and upregulation of SEPT9 in the satellite glial cells of the dorsal root ganglia is associated with blood glucose levels, mechanical threshold, and chronic pain in a rat model of DPN^22^. However, whether SEPT9 is regulated via miR-503-5p in DNP requires further investigation.

In the current study, miR-503-5p was analyzed in the spinal cord of DNP model mice. Additionally, the effects of miR-503-5p on blood glucose, body weight, and tactile responsiveness in DNP model mice were assessed. Finally, we investigated the possible molecular regulatory mechanisms of miR-503-5p in high glucose (HG)-treated astrocytes.

## Methods and materials

### Animals

All the experiments were performed in accordance with the ARRIVE guidelines for reporting animal experiments. For the experiment, 10-week-old BKS-db mice (n = 20) were used as a model of type 2 diabetes mellitus (T2DM), and 10-week-old heterozygous mice (db/m, n = 5) were used as non-diabetic controls. Mice were obtained from GemPharmatech (Nanjing, China). After a 1-week habituation, the db/db mice were randomly assigned to four groups (n = 5): db/db, db/db + NC, db/db + miR-503-5p agomiR, and db/db + miR-503-5p antigomiR. For the administration of miRNA, we administered 5 nmol of miR-503-5p agomiR, 5 nmol of antigomiR, or 5 nmol of miR-NC (miRNA negative control) into the tail vein of db/db mice after anesthesia, as described previously^5^. The injection was administered every four days for a total of four injections. Different functional analyses were performed on the mice two days after the last injection. Finally, mice were euthanized via an intraperitoneal injection of 130 mg/kg of pentobarbital. We ensured that the mice were euthanized by performing cervical dislocation. The L4-L5 spinal cord tissues were isolated and subsequently stored at − 80 °C. The mouse experiments were approved by the Ethics Committee of Quanzhou First Hospital Affiliated to Fujian Medical University (No.2022–215), in accordance with the Basel Declaration.

### qRT-PCR

Total RNA from spinal cord homogenates or astrocytes was extracted using TRIzol reagent (Invitrogen). Total RNA was converted into cDNA using the miRNA 1st-Strand cDNA Synthesis Kit (Takara). PCR was performed using an ABI PCR system (Applied Biosystems). The primer sequences used are as follows: miR-503-5p forward: 5′- ACACTCCAGCTGGGTAGCAGCGGGAACAG-3′, reverse: 5′-CTCAACTGGT GTCGTGGA-3′. U6 forward: 5′-CTCGCTTCGGCAGCACA-3′, reverse: 5′- AACGCTTCACGAATTTGCGT-3′. Fold changes in the transcripts were computed applying the 2^−ΔΔCT^ method and U6 acted as internal reference.

### Body weight, blood glucose, and inflammation

The body weight and blood glucose levels of the mice in each group were measured at 08:00 on the second day after the last injection. Mice were weighed using an electronic scale. Blood glucose in the tails of the mice in each group was detected using a glucometer (Roche, Basel, Switzerland). Inflammation markers including IL-1β, IL-6, and TNF-α in serum and culture supernatant were measured by ELISA.

### Tactile responsiveness with sensitivity to heat

Tactile and heat responsiveness were determined as previously described^5^. For mechanical allodynia assessment, the mechanical withdrawal threshold (MWT) of the sole of the foot of the mice in a chamber with a wire mesh floor was registered using an automatic dynamic plantar aesthesiometer (Ugo Basile, Varese, Italy). MWT was recorded as the minimum force (g) that caused rapid retraction of the right rear paw. This was repeated thrice, and the average force was recorded at 5 min intervals. To assess hypersensitivity to heat algesia, mice were placed in a clear glass panel chamber of 30 ± 1 °C, then a heat source was placed below the back paw, and the latency period of foot thermal withdrawal latency (TWL) and the duration (seconds) between fever onset and foot fever resolution were recorded using a plantar analgesia apparatus (Ugo Basile). This was repeated thrice at 5 min intervals, and the average time was recorded.

### Astrocyte culture with treatment

Astrocytes were isolated from the cerebral cortexes of postnatal day (PND) 2 mice. The cells were soaked in DMEM (Gibco) supplemented with 10% fetal bovine serum (FBS; Gibco). After reaching 95% confluence, dibutyryl cAMP (0.15 mM) was added to the medium to induce differentiation. For the HG treatment, 30 mM glucose was added to the normal culture medium. As a control, astrocytes were cultured in normal glucose concentrations (5.5 mM, NG group). Subsequently, the cells were incubated at 37 °C in an atmosphere containing 5% CO_2_. Reagents including 20 nmol miR-503-5p mimic, 20 nmol miR-503-5p inhibitor, 20 nmol miR-NC, 20 nmol si-SEPT9, 20 nmol si-NC, 50 nmol SEPT9 overexpression construct (ov- SEPT9), and 50 nmol empty pcDNA3.1) vector (ov-NC) were sourced from Shanghai GenePharma. These were transfected into cells using Lipofectamine 2000 (Invitrogen) following the manufacturer’s protocol. All subsequent cell-related detection experiments were independently repeated three times.

### Western blotting

Total protein samples were harvested from the spinal cord homogenates or astrocytes. The protein lysate was loaded onto a sodium dodecyl sulfate–polyacrylamide gel for electrophoresis. After electrophoresis, a PVDF membrane (Millipore) was used to electrotransfer the proteins. After stabilizing, the membranes were soaked in a primary antibody liquid including anti-glial fibrillary acidic protein (GFAP) antibody (1:1000, Abcam), anti-monocyte chemoattractant protein-1 (MCP-1) antibody (1:1000, Abcam), anti-SEPT9 antibody (1:1000, Abcam), or anti-GAPDH antibody (1:1,500, Abcam) at 4 °C overnight. After rinsing, membranes were soaked with HRP-labeled antibody (1:2000, Abcam) for 2 h at 25 °C and then washed. Finally, the protein bands were visualized with enhanced chemiluminescence (Keygentec, Nanjing, China) and ChemiDoc™ XRS systems (Bio-Rad).

### Immunofluorescence

After slightly drying the slide, the histochemical pen was used to circle the evenly distributed cells in the middle of the cover glass to prevent the antibody from flowing away. Then, we added 50–100 μL of membrane-breaking working solution and incubated at room temperature for 20 min, followed by washing with PBS three times, each time for 5 min. Next, 3% BSA was applied evenly to the tissues, which were then sealed and incubated at room temperature for 30 min. A mixed reagent of MCP-1 and GFAP antibodies was added, and the cell culture plate was incubated overnight at 4 °C in a humidified chamber. The cell pore plate was placed on a decolorizing shaker and agitated three times, with each session lasting 5 min. Subsequently, the appropriate secondary antibodies were added and incubated at room temperature for 50 min. The plate was then placed in PBS (pH 7.4) and shaken on the decolorizing shaker for three wash cycles, each lasting 5 min each. After the glass slide was slightly dried, the DAPI staining solution was added dropwise to the circles and incubated at room temperature in the dark for 10 min. The plate was placed in PBS (pH 7.4) and shaken on a decolorization shaker for three wash cycles, each lasting 5 min. After drying the glass slide slightly, it was sealed with an anti-fluorescence quenching sealing agent. Photographs were taken using an inverted fluorescence microscope (Zeiss Spot, Carl Zeiss Ltd., North York, Canada), and the average fluorescence intensity was analyzed using ImageJ.

### Dual-luciferase reporter experiment

The miR-503-5p regulating SEPT9 gene 3′-UTR was forecasted by StarBase 3.0. with TargetScan. The luciferase reporter gene vector (psi-CHECK2) of wild-type (WT) or mutant-type (MUT) SEPT9 mRNA 3′-UTR was formed by Shanghai GenePharma. Briefly, the WT- SEPT9-3′-UTR or MUT- SEPT9-3′-UTR vectors were co-transfected with miR-503-5p mimic or miR-503-5p inhibitor into 239 T cells. In addition, only WT- SEPT9-3′-UTR or MUT- SEPT9-3′-UTR vectors were transfected into 239 T cells as controls (blank group). After 48 h, the luciferase activity was monitored using a Dual-Luciferase Assay kit (Promega). The Renilla luciferase activity was normalized to firefly luciferase activity.

### Hematoxylin and eosin staining

Liver and lung tissues were collected and fixed for sectioning. Hematoxylin and eosin staining was performed according to the manufacturer’s instructions (Beyotime, Shanghai, China).

### Statistical analysis

Data analysis was performed using IBM SPSS version 22.0. The data are presented as means ± standard deviation (SD). Statistical differences between groups (≥ 3 groups) were compared using one-way ANOVA, and least square difference was used for post hoc analysis.* P* values < 0.05 were considered to indicate statistical significance.

### Ethical approval

Mice experiments were approved by the ethics committee of the Quanzhou First Hospital Affiliated to Fujian Medical University (No.2022–215), accordance with the Basel Declaration.

## Results

### miR-503-5p levels decreased and impacted peripheral neuropathy in an in vivo DPN mouse model

**Figure 1:**
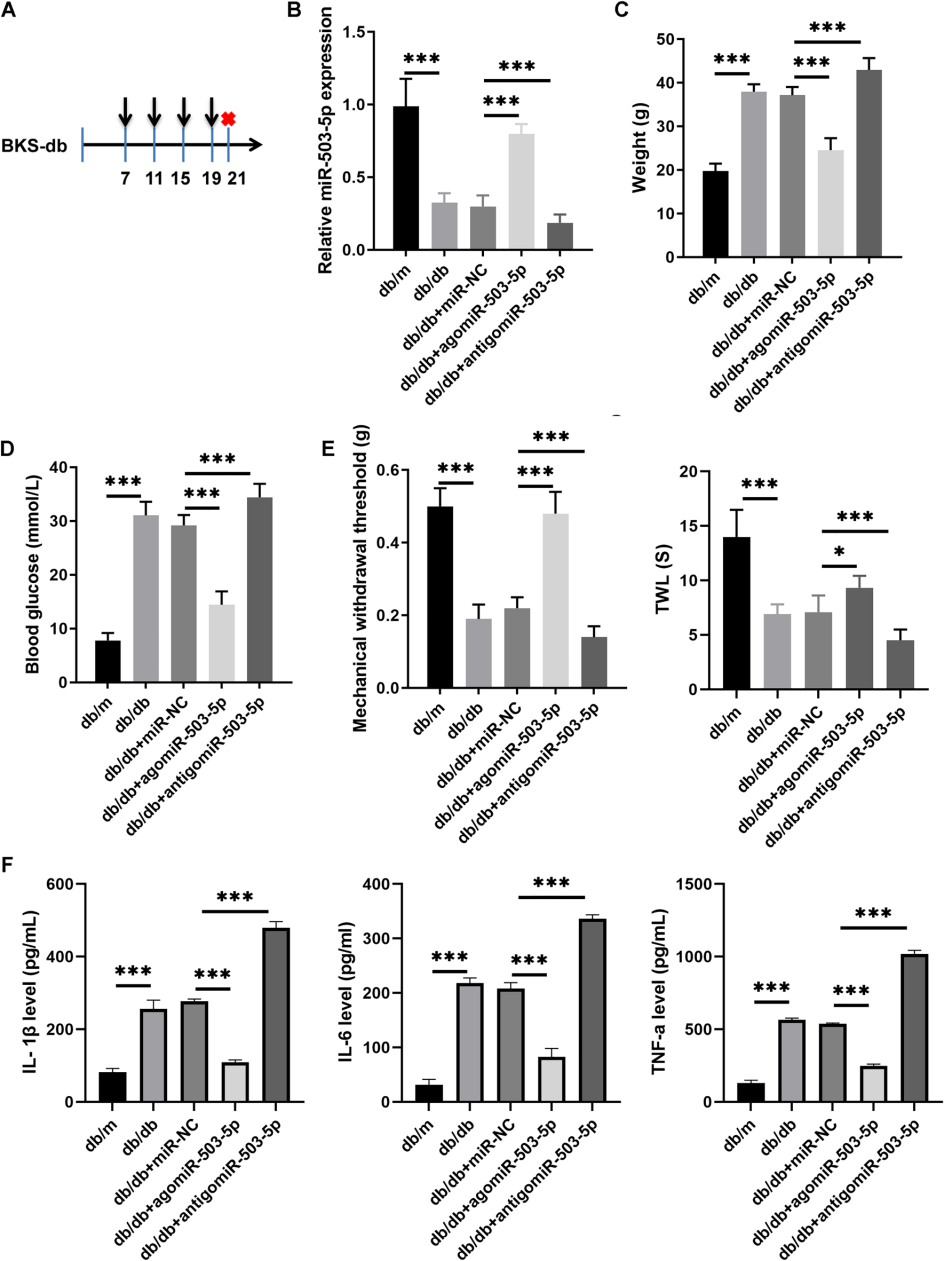
miR-503-5p overexpression reduces the peripheral neuropathy in mice in the Diabetic peripheral neuropathy (DPN) model in vivo. (**A**) Flowchart of the mouse experiment: Mice are adaptively fed for one week before being injected with miRNA, once every four days for a total of four times. Mice are euthanized two days after the final injection. (**B**) The miR-503-5p expression in the spinal cord ofdb/db mice injected with agomiR-503-5p, antigomiR-503-5p, or miR-NC (miRNA negative control) was detected with qRT-PCR. (**C** and **D**) The body weight with blood glucose levels in db/db mice was assessed after injections with agomiR-503-5p, antigomiR-503-5p, or miR-NC. (**E**) db/db mice injected with agomiR-503-5p, antigomiR-503-5p, or miR-NC were assessed by mechanical withdrawal threshold (MWT) and thermal withdrawal latency (TWL). (**F**) The IL-1β, IL-6, and TNF-α levels in serum were measured by ELISA. (*P < 0.05 and ***P < 0.001).

**Figure 2:**
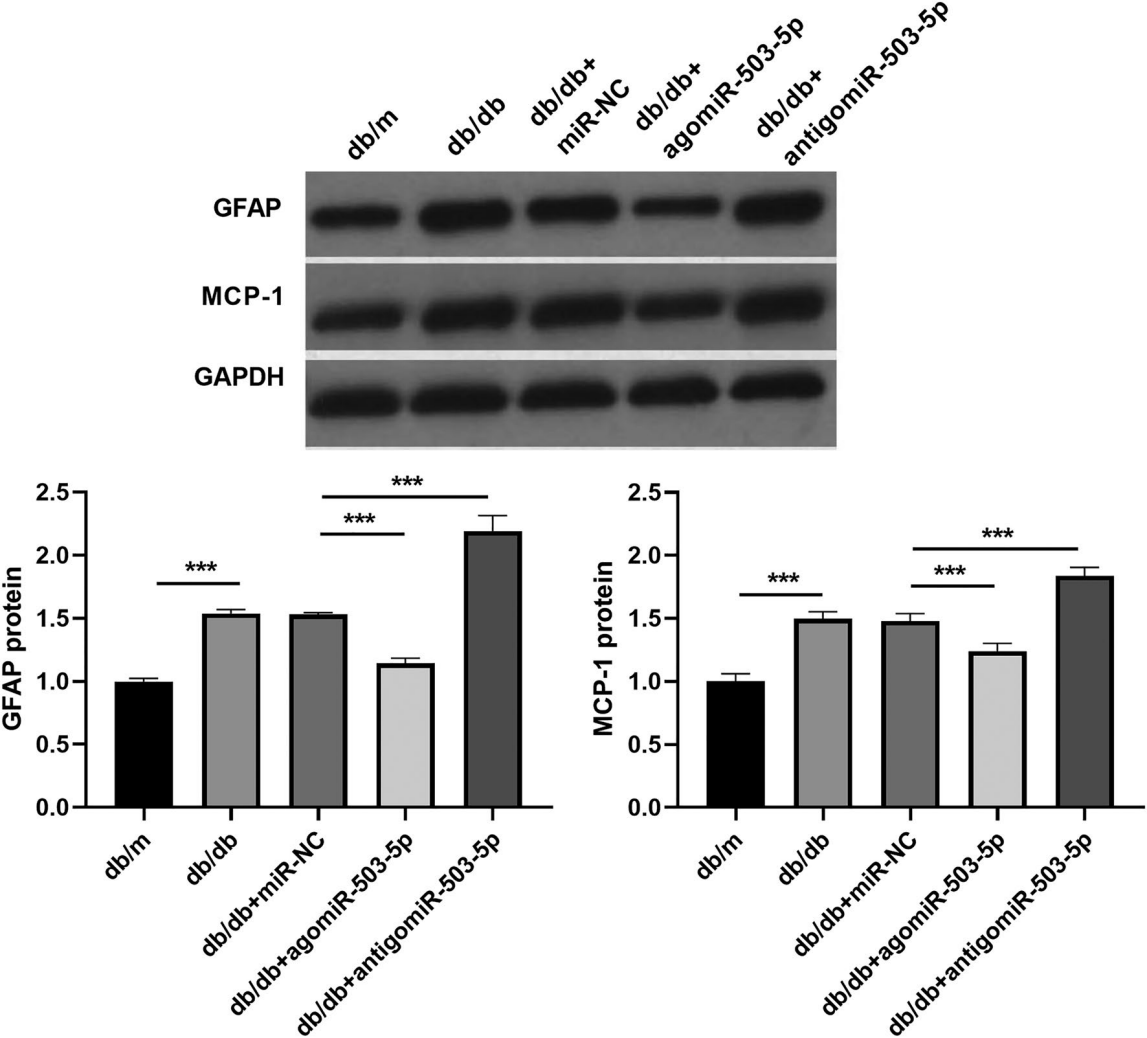
The levels of GFAP and MCP-1 proteins in the spinal cord were regulated by miR-503-5p. The GFAP and MCP-1 protein levels in the spinal cord of db/db mice injected with agomiR-503-5p, antigomiR-503-5p, or miR-NC (negative control) were measured using western blot. Full-length blots are presented in Supplementary Figure 1A (*** P < 0.001).

To investigate the role of miR-503-5p in diabetic peripheral neuropathy, the T2DM mouse model (db/db) was utilized as a severe DPN model, while db/m mice served as controls. The expression levels of miR-503-5p within the spinal cord tissues of db/db mice were then analyzed. A flowchart of the mouse experiments is shown in Fig. 1A. The results showed that miR-503-5p expression decreased in the spinal cord of db/db mice compared to db/m mice (Fig. 1B). In addition, compared with db/m mice, the weight, blood glucose, IL-1β, IL-6, and TNF-α levels increased significantly, while MWT and TWL decreased significantly in db/db mice (Fig. 1C–F). To assess the functional effects of miR-503-5p on peripheral neuropathy in the DPN mouse model in vivo, agomiR-503-5p or antigomiR-503-5p was injected into the tail veins of db/db mice. The expression of miR-503-5p in the spinal cord was significantly elevated in db/db mice treated with agomiR-503-5p, whereas it was notably decreased in db/db mice injected with antigomiR-503-5p, compared to those injected with miR-NC (Fig. 1B). Next, treatment with agomiR-503-5p significantly reduced body weight and blood glucose levels, increased MWT and TWL, and reduced the IL-1β, IL-6, and TNF-α levels compared to db/m mice treated with miR-NC. Conversely, treatment with antigomiR-503-5p exhibited opposite effects (Fig. 1C–F). Compared to db/m mice, the GFAP and MCP-1 protein levels were significantly enhanced in the spinal cord of db/db mice, whereas they were reduced after agomiR-503-5p injection and further enhanced after antigomiR-503-5p injection (Fig. 2). In addition, the injection of agomiR-503-5p and antigomiR-503-5p had no significant effect on the tissue morphology of the liver and lungs (Supplementary Fig. 2), suggesting that no significant side effects were observed after the agomiR-503-5p and antigomiR-503-5p injections.

### MiR-503-5p overexpression or silencing affected the activation of astrocytes with HG

**Figure 3:**
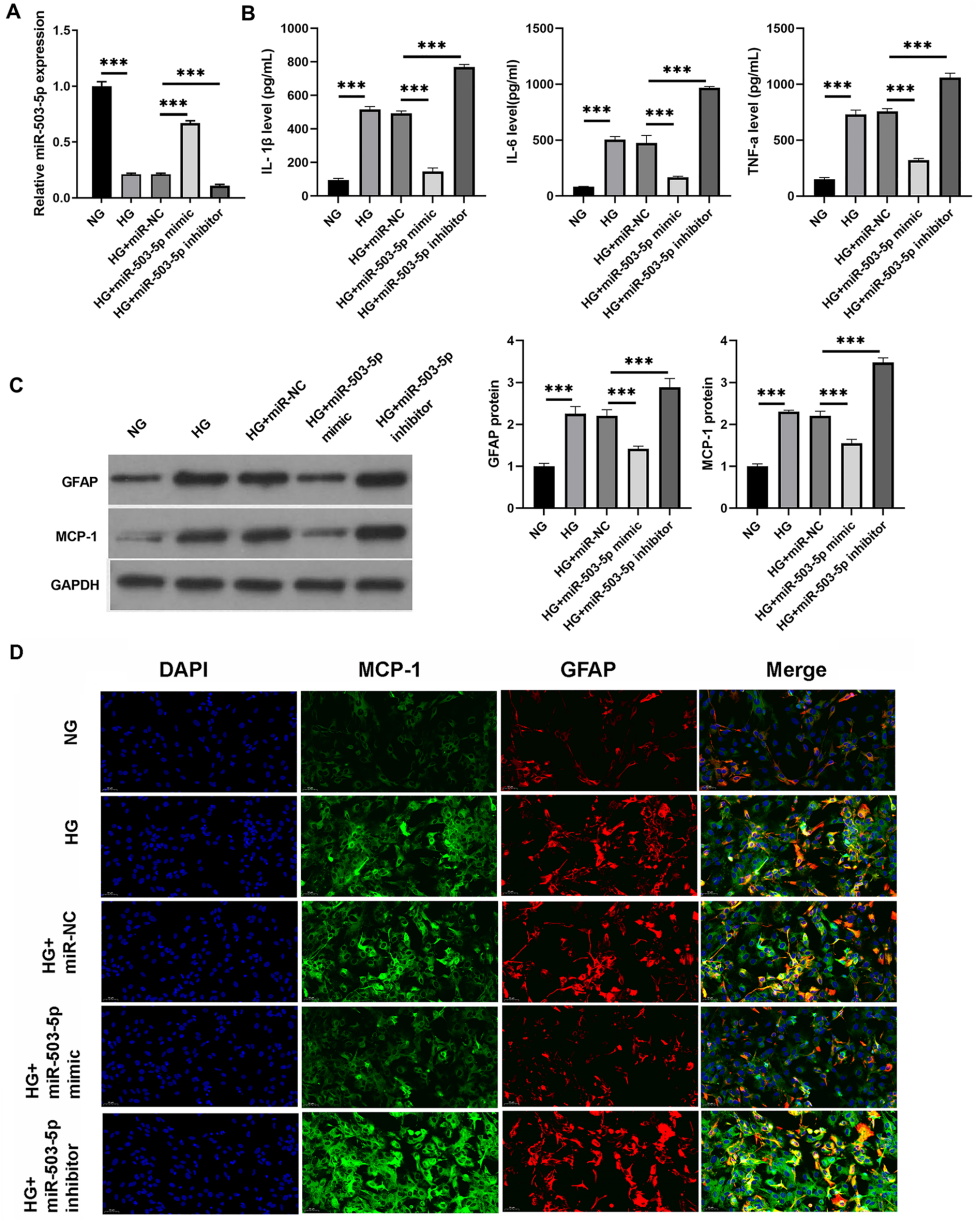
Overexpression and silencing of miR-503-5p influenced the activation of astrocytes treated with high glucose (HG). (**A**) The efficiency of transfection with miR-503-5p mimic or inhibitor in astrocytes exposed to HG was verified using qRT-PCR. (**B**) The levels of IL-1β, IL-6, and TNF-α in the culture supernatant were quantified by ELISA. (**C**) The expression of GFAP and MCP-1 proteins in astrocytes treated with HG following transfection with either miR-503-5p mimic or miR-503-5p inhibitor was evaluated using western blot. Full-length blots are presented in Supplementary Figure 1B. (**D**) The expression of GFAP and MCP-1 proteins in astrocytes treated with HG following transfection with miR-503-5p mimic or miR-503-5p inhibitor was assessed using immunofluorescence (***P < 0.001). For the HG treatment, an additional 30 mM glucose was added to the normal culture medium. NG: Astrocytes were cultured at normal glucose concentrations (5.5 mM).

The role of miR-503-5p in DPN was also assessed. The miR-503-5p mimic or inhibitor was transfected into astrocytes with HG, and qRT-PCR analysis demonstrated that HG remarkably blocked miR-503-5p expression in astrocytes compared to normal cultured astrocytes, whereas the miR-503-5p mimic or inhibitor transfection remarkably overexpressed or silenced miR-503-5p in astrocytes with HG (Fig. 3A). Moreover, HG significantly increased the levels of GFAP and MCP-1 proteins, as well as IL-1β, IL-6, and TNF-α in astrocytes, compared to normal cultured astrocytes. Transfection with miR-503-5p mimic or inhibitor could significantly inhibit or enhance, respectively, the levels of GFAP and MCP-1 proteins and IL-1β, IL-6, and TNF-α in astrocytes treated with HG (Fig. 3B,C). This suggests that overexpression of miR-503-5p could prevent the activation of astrocytes induced by HG, while silencing miR-503-5p had the opposite effect. Similarly, immunofluorescence findings indicated that GFAP and MCP-1 protein levels in the HG group were significantly higher than those in the NG group. Moreover, GFAP and MCP-1 protein levels were significantly reduced in the HG + miR-503-5p mimic group, yet significantly elevated in the HG + miR-503-5p inhibitor group (Fig. 3D and Supplementary Fig. 3).

### SEPT9 was a target of miR-503-5p

**Figure 4:**
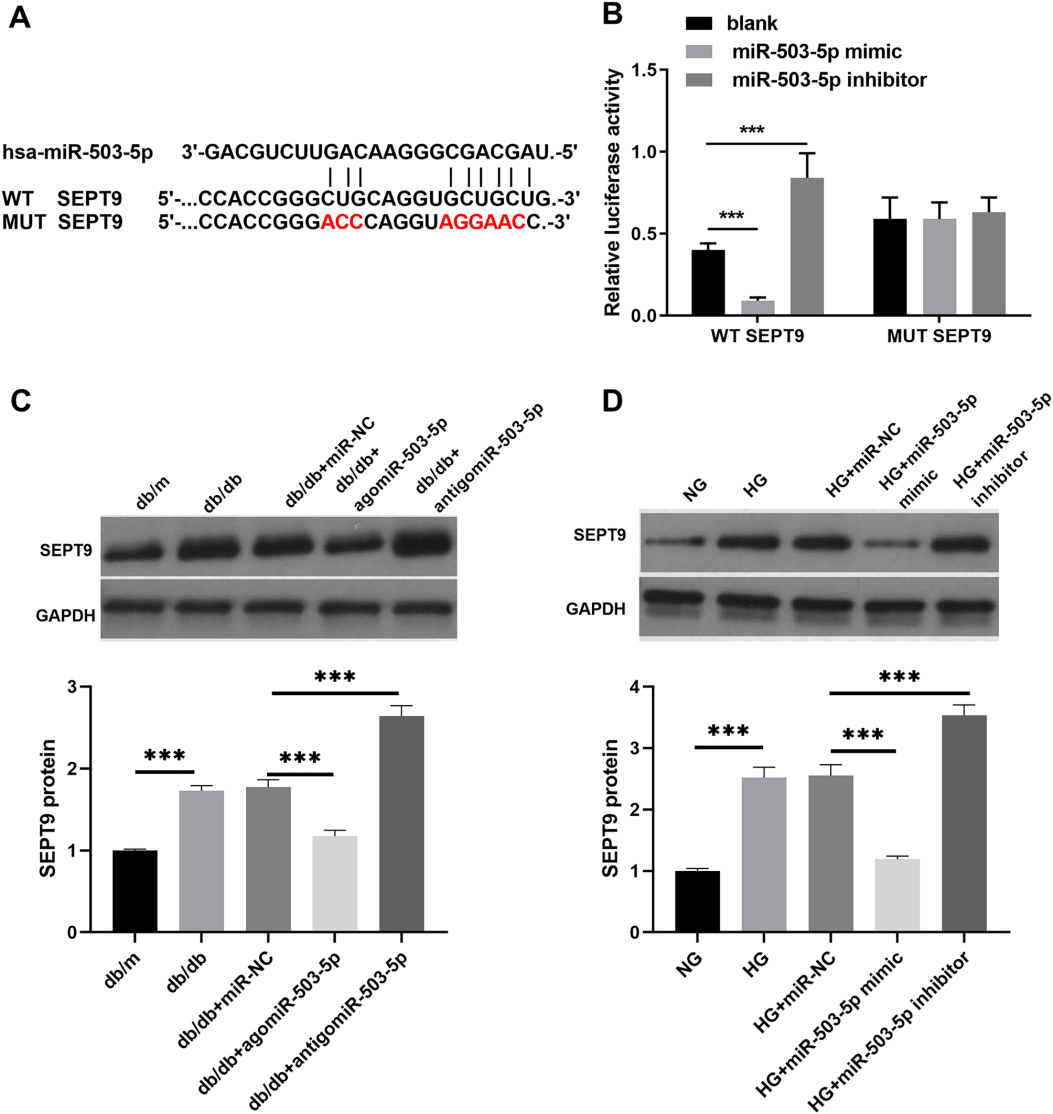
SEPT9 was a target of miR-503-5p. (**A**)The predicted binding site between miR-503-5p and SEPT9. (**B**) The targeted relationship between miR-503-5p and SEPT9 was validated using a dual-luciferase reporter assay. (**C** and **D**) The expression of SEPT9 protein in the spinal cord of db/db mice treated with agomiR-503-5p/antigomiR-503-5p or astrocytes with HG and transfected with miR-503-5p mimic/miR-503-5p inhibitor, was measured using western blot. Full-length blots are presented in Supplementary Figure 1C and D. (***P < 0.001).*WT*: Wild type; *MUT*: Mutant type 3.

The online prediction tool TargetScan with Starbase was used to predict the potential binding target of miR-503-5p. SEPT9 was found to be a potential target of miR-503-5p, with a theoretical binding site in SEPT9 with miR-503-5p (Fig. 4A). For validation, the dual-luciferase assay results showed that the relative luciferase activity decreased by the miR-503-5p mimic but increased by the inhibitor when compared to the blank in the WT-SEPT9 group, whereas the relative luciferase activity by the miR-503-5p mimic or inhibitor made no difference in the MUT-SEPT9 group (Fig. 4B). In addition, compared with the db/db mice, the SEPT9 protein expression was significantly elevated in the spinal cord of db/db mice; compared with the db/db + miR-NC group, the SEPT9 protein expression was significantly reduced in the spinal cords of db/db + agomiR-503-5p mice, while it was enhanced in the spinal cords of db/db + antigomiR-503-5p mice (Fig. 4C). Compared to the NG group, SEPT9 protein expression was significantly increased in HG-treated astrocytes (Fig. 4D). Conversely, compared to the HG + miR-NC group, SEPT9 protein expression was significantly decreased in the astrocytes of the HG + miR-503-5p mimic group and increased in the astrocytes of the HG + miR-503-5p inhibitor group (Fig. 4D).

### SEPT9 overexpression could inverse the suppressive effect of miR-503-5p mimic on astrocytes with HG

**Figure 5:**
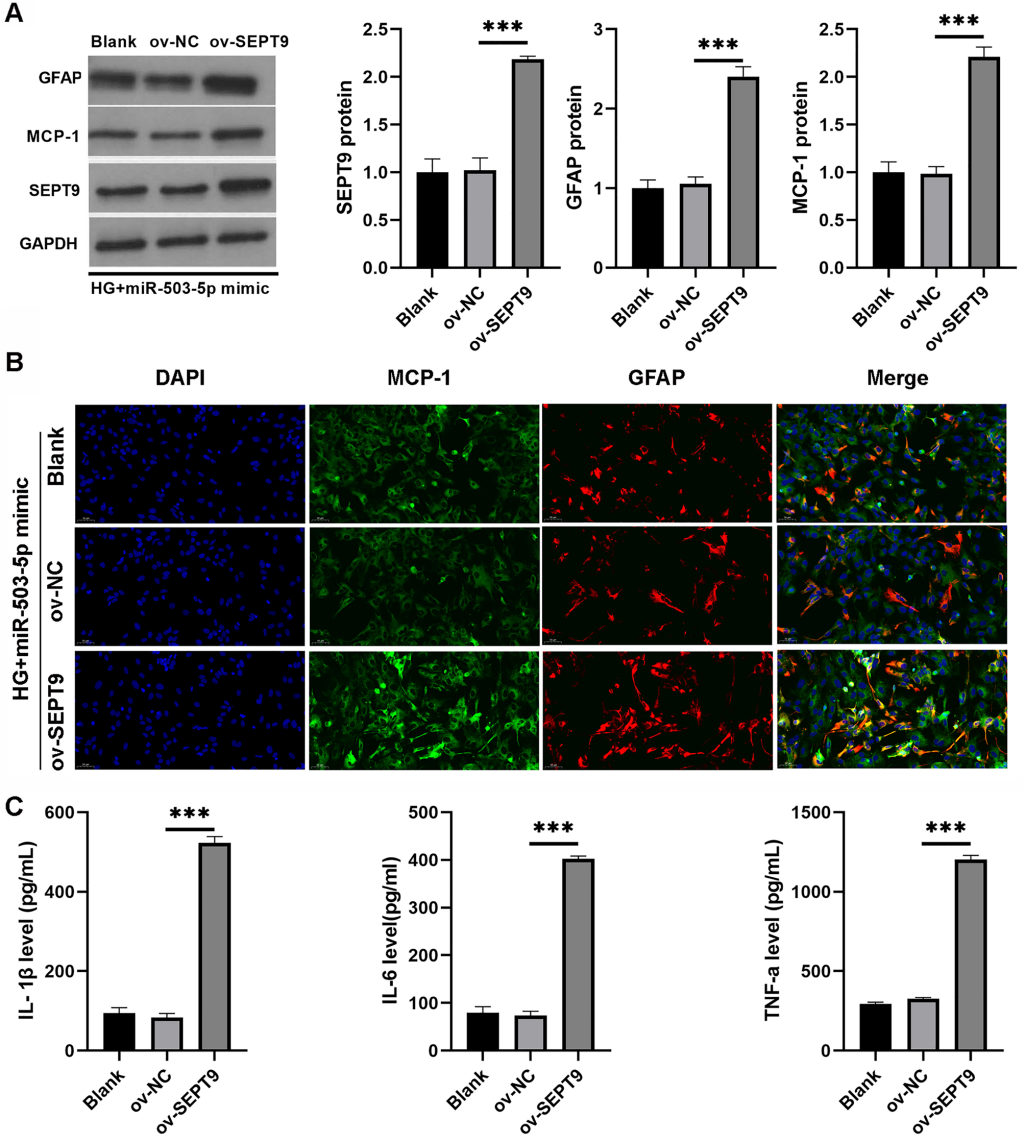
Overexpression of SEPT9 was able to reverse the suppressive effects of miR-503-5p overexpression in astrocytes treated with HG. (**A**) The expression of SEPT9, GFAP, and MCP-1 proteins in astrocytes with HG, following co-transfection with the miR-503-5p mimic and ov-SEPT9, was determined using western blot analysis. Full-length blots are displayed in Supplementary Fig.1E. (**B**) The expression of GFAP and MCP-1 proteins in astrocytes with HG, after co-transfection with the miR-503-5p mimic and ov-SEPT9, was evaluated using immunofluorescence. (**C**) The levels of IL-1β, IL-6, and TNF-α in the culture supernatant were quantified using ELISA after co-transfection with the miR-503-5p mimic and ov-SEPT9. (***P < 0.001).

To determine the effect SEPT9 in astrocytes with HG treatment, the si-SEPT9 was transfected into astrocytes with HG. The results showed that compared with si-NC group, SEPT9 protein was significantly reduced in si- SEPT9 group in HG-treated astrocytes, accompanied by a reduction in MCP-1 protein and the levels of IL-1β, IL-6, and TNF-α (Supplementary Fig. 4). To determine if miR-503-5p mediates its effects in astrocytes with HG through SEPT9, a rescue experiment was conducted by co-transfecting astrocytes with HG with both ov-SEPT9 and a miR-503-5p mimic. Results for overexpression efficiency indicated that SEPT9 protein expression was significantly increased in astrocytes transfected with both ov-SEPT9 and the miR-503-5p mimic (Fig. 5A). Furthermore, the expression of GFAP and MCP-1 proteins was significantly higher in astrocytes transfected with ov-SEPT9 plus the miR-503-5p mimic under HG conditions compared to those transfected with ov-NC plus the miR-503-5p mimic (Fig. 5A). Immunofluorescence analysis also revealed a significant increase in MCP-1 and GFAP proteins in astrocytes transfected with ov-SEPT9 and the miR-503-5p mimic (Fig. 5B and Supplementary Fig. 5). Levels of IL-1β, IL-6, and TNF-α were significantly higher in astrocytes treated with HG and transfected with ov-SEPT9 plus the miR-503-5p mimic compared to those treated with HG and transfected with ov-NC plus the miR-503-5p mimic (Fig. 5C).

## Discussion

The prevention of DPN remains challenging owing to its complex pathogenesis. Recent studies have implicated miRNAs in DPN^23^. Therefore, we investigated the mechanism underlying the effects of miR-503-5p on DPN. Herein, we confirmed that miR-503-5p expression was reduced in the spinal cord of DPN mice and astrocytes subjected to HG. Tail vein injections of agomiR-503-5p markedly alleviated peripheral neuropathy-induced neuropathic pain in T2DM mice, while antigomiR-503-5p had the opposite effect. MiR-503-5p mimic or inhibitor markedly blocked or elevated the MCP-1 expression and activation of astrocytes with HG. MCP-1 secreted by activated astrocytes enhances pain sensitivity and promotes persistent pain states^24^. SEPT9 was a target miR-503-5p. The decrease in SEPT9 is functionally similar to miR-503-5p overexpression in HG-treated astrocytes. Furthermore, SEPT9 mimic disrupted the repressive effect of miR-503-5p overexpression in astrocytes treated with HG. These results suggest that miR-503-5p in astrocytes can alleviate peripheral neuropathy-induced neuropathic pain in mice with DPN by regulating SEPT9, providing theoretical support for miR-503-5p as a promising biomarker for the clinical treatment of DPN.

Recently, numerous studies have reported that multiple miRNAs are aberrantly expressed in DM and related complications and play a dynamic role in gene regulation^25^. Similar to the findings of Hu et al.^26^, miR-34c was found to modulate DPN by regulating autophagy. miR-30d-5p is increased in DM mouse neurons, while miR-30d-5p attenuates DPN by modulating SIRT1 expression^27^. There are many studies on the role of miR-503-5p in different cancers, but few studies have examined its role in DM and its related complications. In diabetic cardiomyopathy and foot syndrome, the expression of miR-503-5p is significantly upregulated^14,15^. Contrary to previous studies, this project shows that miR-503-5p expression is down regulated in DPN. In addition, miR-503-5p promoted inflammation and oxidative stress in high glucose-induced microvascular endothelial cell injury^17^. Conversely, miR-503-5p overexpression in astrocytes could inhibit inflammation and alleviate peripheral neuropathy in DPN mice. These studies indicates that the function of miR-503-5p may play different roles in different complications of diabetes.

MiRNAs negatively regulate target gene expression^28^. In this study, we found that SEPT9 is a target of miR-503-5p. SEPT9 is implicated in the neural pain of hereditary neuralgic muscular atrophy^21,29^. Furthermore, SEPT9 expression is increased in satellite glial cells associated with the DPN rat model, which associated with blood glucose levels, mechanical threshold, and chronic pain in a rat model of DPN^22^. Similarly, SEPT9 protein was elevated in db/db mice and astrocytes treated with HG, which promoted neural pain and attenuated the repressive effect of the miR-503-5p. These results suggested that miR-503-5p overexpression in astrocytes could alleviate peripheral neuropathy in DPN mice via SEPT9 regulation.

In conclusion, miR-503-5p overexpression in astrocytes alleviated peripheral neuropathy-induced neuropathic pain in DPN mice by regulating SEPT9. MiR-503-5p may be a novel therapeutic molecular target for DPN.

### Supplementary Information

Supplementary Information 1.

Supplementary Information 2.

## Data Availability

The data used or analysed during the current study are available from the corresponding author on reasonable request.
