# Supplementary material for: MiR-503-5p alleviates peripheral neuropathy-induced neuropathic pain in T2DM mice by regulating SEPT9 to inhibit astrocyte activation
Source: Sci Rep. 2024 Jun 21;14:14361. doi: 10.1038/s41598-024-65096-z (PMC11192719; doi:10.1038/s41598-024-65096-z)
Supplement: Supplementary file 1 — Supplementary Information 1. [file 41598_2024_65096_MOESM1_ESM.docx]

**Supplementary Table 1 Sequences**

| miRNA/gene | Sequence (5′–3′) |
| --- | --- |
| miR-503-5p agomiR | UagcagcgggaacagUacUgcag |
| miR-503-5p-antigomiR | aUcgUcgcccUUgUcaUgacgUc |
| miRNA negative control | UCACAACCUCCUAGAAAGAGUAGA |
| miR-503-5p mimic | UagcagcgggaacagUacUgcag |
| miR-503-5p inhibitor | CUGCAGUACUGUUCCCGCUGCUA |
| miR-NC | UCACAACCUCCUAGAAAGAGUAGA |
| si-SEPT9 | agaagcgcaUUcccgacacccgdTdT |
| si-NC | UUCUCCGAACGUGUCACGdTdT |
