# Supplementary material for: MiR-503-5p alleviates peripheral neuropathy-induced neuropathic pain in T2DM mice by regulating SEPT9 to inhibit astrocyte activation
Source: Sci Rep. 2024 Jun 21;14:14361. doi: 10.1038/s41598-024-65096-z (PMC11192719; doi:10.1038/s41598-024-65096-z)
Supplement: Supplementary file 2 — Supplementary Information 2. [file 41598_2024_65096_MOESM2_ESM.pdf]

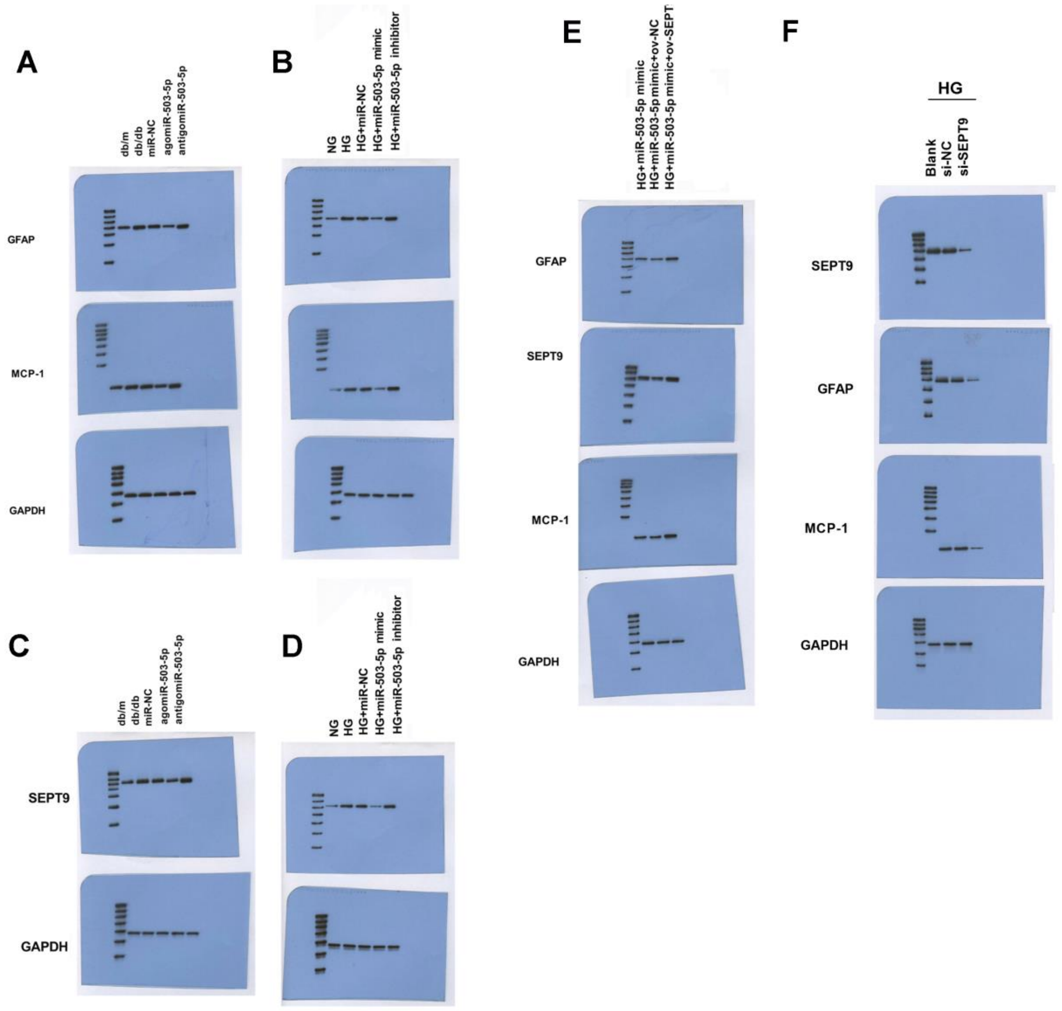

Supplementary Figure 1 The whole uncropped images of the original western blots.

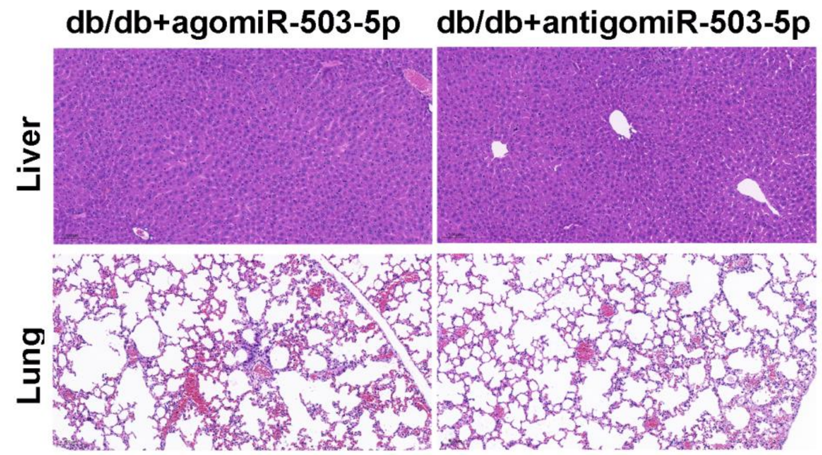

Supplementary Figure 2 The injection of agomiR-503-5p and antigomiR-503-5p has no significant effect on the tissue morphology of the liver and lungs.

The tissue morphology of the liver and lungs were analyzed by HE stain (Magnification: 100

times).

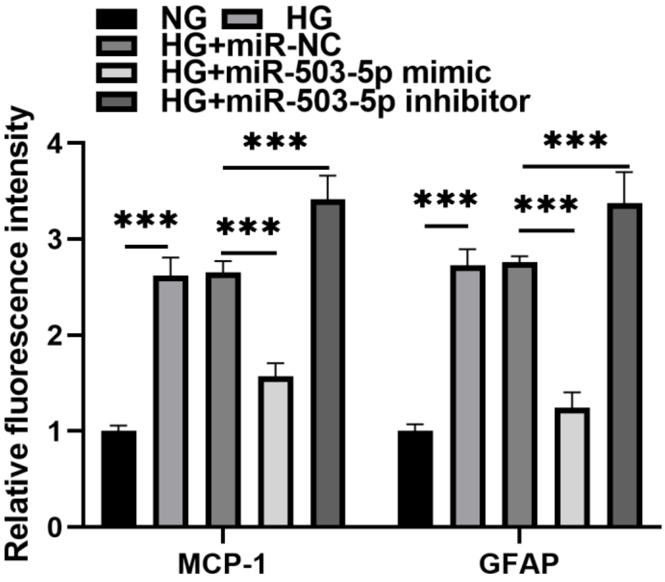

Supplementary Figure 3 The relative fluorescence intensity in astrocytes after miRNA transfection is shown. NG: astrocytes were cultured at normal glucose concentrations (5.5 mM).HG: astrocytes were cultured at high glucose concentrations (30.0 mM). miR-NC, miR-503-5p mimic, and miR-503-5p inhibitor groups represents miR-NC, miR-503-5p mimic, and miR-503-5p inhibitor transfection. (*P<0.05 and ***P<0.001).

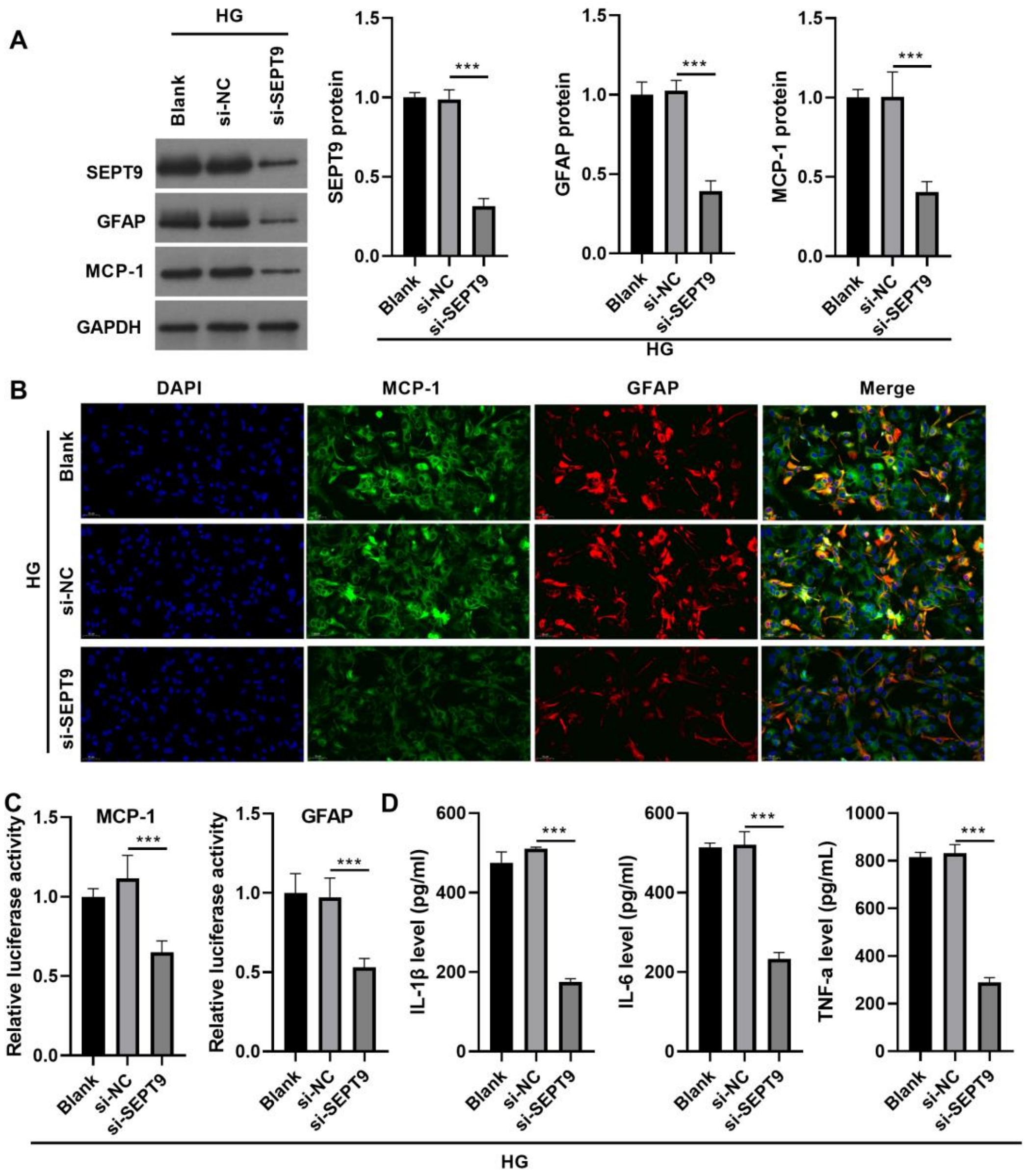

Supplementary Figure 4 Silencing of SEPT9 inhibited the activation of astrocytes treated with high glucose (HG). (A) SEPT9 and MCP-1 in astrocytes exposed to HG was verified using western blot after si-SEPT9 transfection. Full-length blots are presented in Supplementary Figure 1F. (B and C) The expression of GFAP and MCP-1 proteins in astrocytes treated with HG following transfection with si-SEPT9 was assessed using immunofluorescence. The relative fluorescence intensity in astrocytes after si-SEPT9 transfection is shown. (D) The levels of IL-1β, IL-6, and TNF-α in the culture supernatant were quantified by ELISA after si-SEPT9 transfection. (D) (****P* < 0.001). For the HG treatment, an additional 30 mM glucose was added to the normal culture medium.

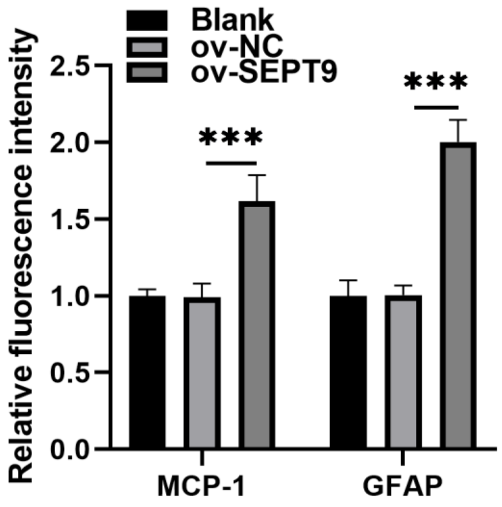

Supplementary Figure 5 The relative fluorescence intensity in astrocytes after ov-SEPT9 transfection is shown. Astrocytes were cultured at high glucose concentrations (30.0 mM). Blank: No plasmid transfection; ov-NC: Transfection of empty plasmids; ov-SEPT9: Transfection of ov-SEPT9 plasmids. (****P*<0.001).
